# Round the Hospitals

**Published:** 1917-01-06

**Authors:** 


### ROUND THE HOSPITALS.

In this, our first issue for 1917, we wish every matron, sister, and nurse, and all workers among the sick, from the highest to the lowest, a Blessed New Year. The closing weeks of the Old Year have been full of good work for British nurses and their profession. The Hon. Arthur Stanley has worked like a Trojan in his endeavour to make it clear exactly what the College of Nursing will do for nurses and their profession. The time has arrived, in our judgment, when a meeting of all nurses who have registered with the College should be summoned, to enable Mr. Stanley to put forward a clear manifesto, pulverising the false statements and misrepresentations which have been sown by the miscalled friends of the Central Committee. This Committee has steadily lost ground, credit, and caste by the policy adopted by those who are striving to keep it alive for their own purposes. Before January is out, providing Mr. Stanley, the Parliamentary leader of the British nursing profession, assembles such a meeting of graduate and registered nurses, and publicly burns the tares which the enemy has sown, he will put an end, for practical purposes, to any influence or authority possessed by the few agitators who have produced disunion for their own ends.

---

The course is clear for the disinterested friends of British nurses who support the College of Nursing, Nurse Registration, and the Statutory Recognition and Defence of the profession. British nurses everywhere are displaying courage, resolution, and awakenment to their own interests. They are coming forward in self-defence in increasing numbers every day. Probably the most remarkable proof of unity and determination British nurses could have given is the fact, that in less than one month, at the request of the Editor of *The Nursing Mirror*, one thousand nurses, properly, have felt the occasion so important as to prepare the necessary documents and complete their papers for registration with the College. The importance of the evidence of unity and decision thus afforded by British nurses cannot be exaggerated, seeing that this spontaneous and remarkable demonstration of the mind of the British Nursing Profession has been manifested in the busiest weeks of the nursing year. British nurses by this action have expressed in the most convincing and practical manner the wish and will of the overwhelming majority of British nurses in Great Britain.

---

British nurses, having commenced 1917 with this magnificent demonstration of unity and purpose to secure for their profession a Royal College of Nursing, with State Registration and Statutory Recognition and Protection, the moment has arrived for all who have influence and associations with trained nurses to join hands, close the ranks, and co-operate in an united effort to bring 30,000 nurses to register with the College by the end of February. On the latest average British nurses are at present joining the College Register at the rate of sixty-five per diem. All that is required is to increase this number to 300 per diem, to accomplish this enrolment of the New Army of British Nurses in support of their Parliamentary Leader the Honourable Arthur Stanley, C.B., M.P. Then Mr. Stanley will be able, we are confident, to do his part with the cordial good will and co-operation of Parliament and the nation. British nurses have done, and are doing, magnificent service, not only at home but wherever the Allies are fighting the battle of freedom and emancipation for the smaller nations, and, indeed, for all civilisation and justice. This being so, there is a paramount opportunity for every matron, sister, and nurse, and for all who are associated with them in their working life, to join hands and make it their immediate and continuous business, to place the names of 30,000 British nurses on the College Register by February 28, 1917.

---

The only danger ahead at the moment is the "wait and see" policy favoured by the timid, inexperienced, well-meaning, but weak people for whom the present reorganisation and uplifting of the British Nursing Profession is too great an enterprise for them to handle. We are proud to know from demonstrable evidence that we represent not only the disinterested friends of British Nurses, but of all that is best in British nursing to-day. The hour for action has arrived. We have confidence that amongst those on the roll of certificated nurses of every hospital where nurses are trained throughout Great Britain, there are able women who will gladly lend a hand by first registering with the College themselves, and then giving their services as Recruiting Sergeants to provide that their sister nurses on the Roll shall have immediate opportunity to fill in an application form and register with the College. But what of the matrons and sisters throughout the land, with their influence and special knowledge? Will not they become Colonels and Captains in the New Army of Registered Nurses who, led by the Hon. Arthur Stanley, are out to secure for the profession of British nurses all that will make it the greatest and best organisation of the kind the world has yet seen? We have many hundreds of friends amongst them. We ask them to join hands once again, as they have done in the past, by procuring from Miss Rundle the necessary application forms for registration with the College, or by requesting us to send them the number they may require, which the Editor will be glad to do by return of post. Some of them may be able and willing to give direct personal service by co-operating with *The Nursing Mirror*, and agreeing to attend a reception of the nurses in The Hospital Building on some Monday or Thursday convenient to them during the next two months. They will find particulars in *The Nursing Mirror* of the 6th instant, and if they will send us a postcard as to what personal service they are prepared to give, we shall welcome their invaluable co-operation.

(*Continued on page* 291.)

### ROUND THE HOSPITALS.

(*Continued from page* 286.)

We urge all interested to believe and remember that if all will put their shoulder to the wheel now and continuously for the next two months they will secure the triumph of British nursing. To co-operate to this extent will make their profession the strongest and best organised of all. We have done our best to help, and speedy victory is assured providing every person of disinterested good will, connected with the nursing profession or interested in it, will do theirs. "Push and Go," not "Wait and See," is the policy which will win triumph for British Nurses and their Profession.

Very properly criticism has been levelled at the College of Nursing, Limited, because the conditions of registration, for the purpose of admitting practising nurses to the Register of the College, which it circulated in October, provided that those Poor-Law infirmaries are recognised for training in which there are not fewer than 250 beds. The Council of the College have now had these regulations (5 *a* and *c*), as published, under their consideration, with the result that they have added a fourth sub-section (*d*) which reads: "or such schools as are recognised at the present date, December 1916, by the Local Government Board as training schools." This new sub-section will apply "during the period of grace granted to existing nurses." The effect of the new sub-section will be to bring in thirteen Poor-Law institutions, which would not otherwise have come in under the old "Beds" regulations. We are glad to have an opportunity to publish these necessary modifications of No. 5 of the "Conditions of Registration applicable to Existing Nurses during the period of grace." They demonstrate the genuine determination of the Council of the College to meet every legitimate claim put forward in the interests of each and all sections of the Nursing profession.

